# Selecting a comparison group for 5-year oral and pharyngeal cancer survivors: Two methods

**DOI:** 10.1186/1471-2288-12-63

**Published:** 2012-05-02

**Authors:** Henrietta L Logan, Scott L Tomar, Myron Chang, Glenn E Turner, William M Mendenhall, Charles E Riggs

**Affiliations:** 1 Department of Community Dentistry and Behavioral Science, University of Florida, 1329 SW 16th Street Room 5174, Gainesville, FL, 32610-3628, USA; 2 Department of Biostatistics, University of Florida, Gainesville, FL, USA; 3 Department of Prosthodontics, University of Florida, Gainesville, FL, USA; 4 Department of Radiation Oncology, University of Florida, Gainesville, FL, USA; 5 Department of Medicine, University of Florida, Gainesville, FL, USA

## Abstract

**Background:**

To assess potential long-term consequences of cancer treatment, studies that include comparison groups are needed. These comparison groups should be selected in a way that allows the subtle long-range effects of cancer therapy to be detected and distinguishes them from the effects of aging and other risk factors. The purpose of this investigation was to test two methods of recruiting a comparison group for 5-year oral and pharyngeal cancer survivors (peer-nominated and listed sample) with emphasis on feasibility and the quality of the match.

**Methods:**

Participants were drawn from a pool of 5-year survivors treated at a large Southeastern hospital. A peer-nominated sample was solicited from the survivors. A listed sample matched on sex, age, and zip code was purchased. Telephone interviews were conducted by a professional call center.

**Results:**

The following represent our key findings: The quality of matching between survivors and listed sample was better than that between survivors and peer-nominated group in age and sex. The quality of matching between the two methods on other key variables did not differ except for education, with the peer method providing a better match for the survivors than the listed sample. The yield for the listed sample method was greater than for the peer-nominated method. The cost per completed interview was greater for the peer-nominated method than the listed sample.

**Conclusion:**

This study not only documents the methodological challenges in selecting a comparison group for studies examining the late effects of cancer treatment among older individuals but also documents challenges in matching groups that potentially have disproportionate levels of comorbidities and at-risk health behaviors.

## Background

Individuals are surviving cancer for longer time periods [1,2]. However, survivors face treatment protocols that may produce late effects that negatively affect quality of life [3-5]. Some effects appear transient and end with treatment [6], but many may persist for months and even years [3,7-9]; others develop later [10,11]. The greatest collection of work on long-term treatment effects is related to childhood cancers but evidence on adult-onset malignancies is accumulating (e.g., [3,4,12-18]). Few cancer treatments are free of risks and most survivors face long-term adverse consequences of treatment [3]. Despite advances in our current cancer survivorship research, there are still serious gaps in our knowledge of late effects, especially in understudied cancers of older individuals [19].

Investigation of older cancer survivors to identify and document the prevalence of these late and long-term effects requires careful attention to the selection of an appropriate comparison group [20]. For instance, as people age, medical comorbidities become more common. Among individuals with high lifetime exposure to alcohol and tobacco, the proportion developing comorbidities, including cardiovascular disease, is believed to be greater than in the general population [21]. In studying the rate at which late effects from cancer treatment occur, including conditions such as cardiovascular diseases or secondary tumors, researchers must be careful that the effect of the cancer treatment is not being confounded with the effects of aging or “at-risk” health behaviors.

The study of late effects of treatment among oral and pharyngeal cancer (OPC) survivors presents challenges similar to those of other cancers in older individuals [22]. Many survivors of OPC face disfiguring surgery, damage to oral function, and increased acute and late toxicities resulting from more aggressive multimodal treatment regimens [23]. In addition, these cancers disproportionately affects older ethnoracially diverse men for whom accurate incidence data on specific negative health outcomes may not be available [24]; there are also no normative data for frequently used assessment scales [25]. In the past, few studies of OPC survivors included a control group matching the target group on relevant lifestyle factors, such as tobacco and alcohol use [20]. This limitation in otherwise informative studies makes it problematic to draw conclusions about the long-term effects of cancer therapy [25-28].

Several methods to produce comparison data have been proposed, ranging from community-based to within-subject designs [29]. One major problem with population-based comparison groups is that tobacco and alcohol lifetime exposure may be lower than those of cancer survivors. This is a particular problem in the case of OPC because tobacco and alcohol use are putative risk factors and independently produce negative health outcomes. Therefore, matching on tobacco use and alcohol use is desirable if the effects of treatment are to be separated from those associated with these “at-risk” behaviors [30].

A peer-matched strategy is believed to offer advantages [31-33]. Compared to the case-survivors, friends may use the healthcare system in similar ways and may be of similar socioeconomic background and lifestyle [34]. Tobacco and alcohol use may also be similar. Older survivors, however, may not be able to nominate a peer, especially when they have experienced debilitating late effects and have become socially isolated [35]. Concern is often raised that using friend-nominated controls may result in overmatching, a phenomenon in which matching occurs on an intermediate variable in a causal pathway resulting in bias [36]. When the risk factors for the disease are age and lifestyle-related and we seek to examine the consequences of cancer treatment, it may be necessary to homogenize the group [37,38].

The purpose of this investigation was to compare and contrast two methods of recruiting a comparison group for five-year survivors of OPC—a peer-nominated control group and a listed sample—on quality of the match, costs, and feasibility. Match was defined as similarity in sex, race, age, smoking and alcohol use, employment, and education level [39].

## Methods

A professional call center conducted a 20-minute telephone interview with all participants in this project. The overall goal of the interview was to assess pain levels of a matched non-cancer comparison group to 5-year survivors of head and neck cancer and to test predictive models of oral pain among 5-year survivors. The survey and methodology received prior approval from the Institutional Review Board at the University of Florida. Items were drawn from published instruments and are reported elsewhere [40]. Participants received a $25 gift card for completing the survey.

A target of 100 survivors, 100 friends, and 100 from the listed sample had been set in advance. Best practices (to meet the target numbers) were used by the professional call center to contact participants for each of the three groups, cancer survivor group, peer-nominated comparison group, and listed sample comparison group.

### Selecting survivors

**Figure 1:**
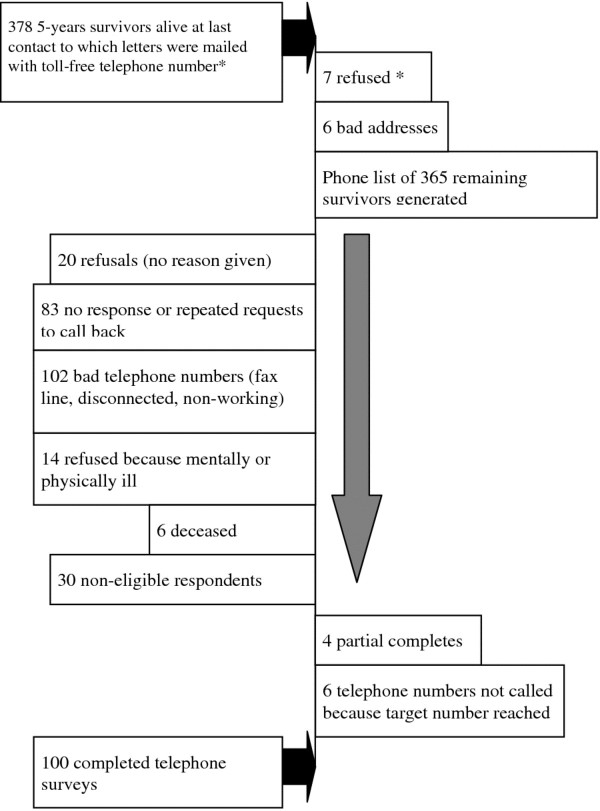
Flowchart of survivor recruitment.

Survivors were drawn from 378 individuals treated for OPC at the institution’s radiation oncology clinic and who had survived five years (plus or minus three months). After verification of status and contact information, the 356 remaining individuals were sent a letter describing the study. (See Figure 1.) A toll-free number was provided for those who did not wish to participate in the study, and seven individuals requested not to be called for the survey. The subsequent list was turned over to the call center who conducted the survey.

### Selecting peer-nominated group

**Figure 2:**
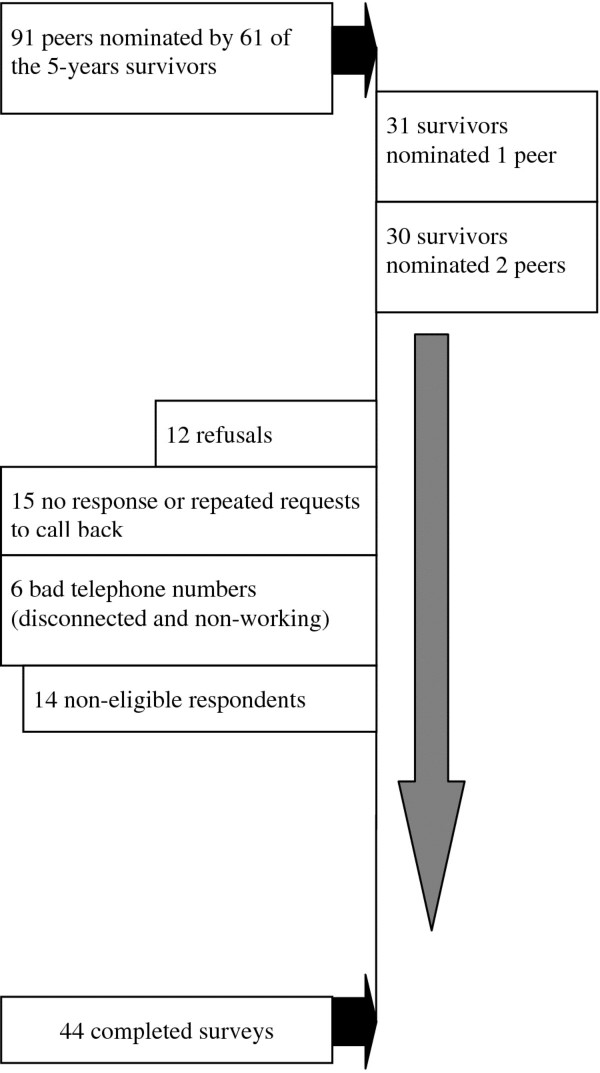
Flowchart of peer nominee recruitment.

Individuals from the survivor group were contacted first and were asked to provide the name and telephone number of two peers who were similar to themselves, who had not had cancer, and who might participate. Peers were screened to exclude respondents with a history of cancer. (See Figure 2)

### Selecting listed sample methodology

**Figure 3:**
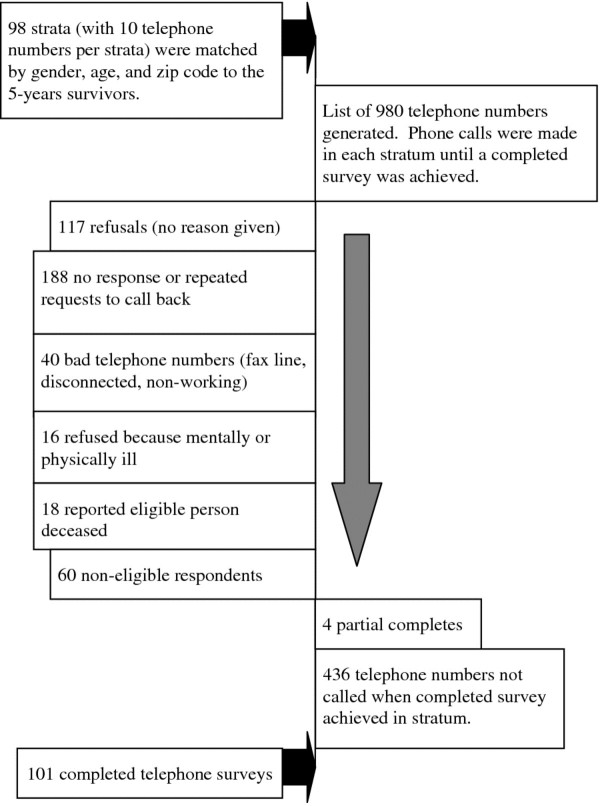
Flowchart of listed sample recruitment.

A commercial list was purchased that matched the survivors by age (within five years), sex, and geographical location. Ten people of the same sex and age (+/− five years) were selected from each survivor’s zip code. Numbers were chosen randomly from that list until one individual completed the survey. Each participant was screened to exclude those with a history of cancer. (See Figure 3)

### Data analysis

Descriptive statistics were calculated as percentages, means, and standard deviations. To calculate the cost incurred, we divided the base cost of $25/hour, which included both fixed and variable costs for the survey, by the number of surveys completed per hour for each group respectively. This base cost may have differed regionally but provided general guidance for cost comparison. That amount was multiplied by the number of completed surveys for each method, giving a measure of total costs per group.

We evaluated the matching properties of the samples onsex, race, age, education, employment status, and cigarette and alcohol use. Alcohol use was derived from these questions: “How often do you drink?” “How much do you drink when you drink?” and “How often do you drink at least six drinks in one day?” Duration of smoking was calculated as the difference between age at the first cigarette and age at last cigarette. For current smokers, current age was used as age at last cigarette.

Two analytic strategies were used. First, we tested for marginal group differences. We then tested for the quality of the match at an individual pair level.

To test for marginal group differences in each variable, we used two-sided p-values for testing H_o_: “the marginal distribution of survivors is the same as that of peers” vs. H_a_: “the marginal distributions are different.” Similarly, we used two-sided p-values for testing H_o_: “the marginal distribution of survivors is the same as that of listed sample” vs. H_a_: “the two marginal distributions are different”; the smaller the p-value, the larger the difference between the two marginal distributions.

To compare the quality of match between the survivor and the peer to that between the survivor and the listed sample, we calculated the absolute difference between each pair on each variable. We used a Wilcoxon rank sum test to determine whether the two groups of differences were different. One-sided p-values were reported for testing H_o_: “the quality of matching between peers and survivors is the same as that between listed sample and survivors” vs. H_a_: “the quality of matching between peers and survivors is better than that between listed sample and survivors.” If p-value < 0.05, then we would claim that the quality of matching between peers and survivors was better than that between listed sample and survivors; if p-value > 0.95, then vice versa. If p-value < 0.5, then there was a trend that the quality of matching between peers and survivors was better than that between listed sample and survivors; if p-value > 0.5, then vice versa.

We used the Wilcoxon rank sum test to compare the locations of two distributions. Because the data contained paired and unpaired observations, the assumption of independence between observations did not hold. The method proposed by Hollander and colleagues [41] was adapted for correlated data. P-values were obtained from testing the group difference by the permutation method described in Dallas and Rao [42].

We compared both marginal group differences and absolute differences between pairs in order to provide a comprehensive picture to evaluate each method. For instance, we expected the match on age and sex to be excellent for the listed sample and survivors because the telephone screening specified this match. If the quality of the match was similar for the peer group and survivors on these variables, then we could conclude that either approach was adequate. In contrast, we might expect alcohol and tobacco use to be more similar between the individuals in the peer group and the survivors than between those matched in the listed sample. The comparison of two marginal distributions may, however, be misleading. For example, the paired data (1,10), (10,1), (1,10), (10,1) show no difference between the two marginal distributions (means equal 5.5), but the quality of match at the individual level is quite poor.

## Results

### Characteristics

Table 1 shows subject characteristics divided by selection method. Table 2 shows the means and Table 3 shows the p-values for matching variables (including sex, age, education, employment, smoking and alcohol history).

**Table 1:** Subject characteristics by selection methods

**Characteristic**	**Survivor N = 100**	**Peer N = 44**	**Listed Sample N = 101**
Sex of subject			
Men	72%	43%	71%
Women	28%	57%	29%
Race			
White	94.0%	96.0%	93.0%
Black	2.0%	2.0%	4.0%
Other	4.0%	2.0%	3.0%
Education			
Less than HS	7.0%	6.8%	10.9%
High School grad			
or equivalent	21.3%	25.0%	21.8%
Post HS education	71.6%	68.2%	67.4%
Employment Status			
Currently employed	32.0%	50.0%	43.0%
Retired	68.0%	47.0%	57.0%
Other	-	3.0%	-
Disabled	19.0%	4.8%	10.3%
Married	72.0%	77.3%	79.2%
*Prevalence of current smoking	15.0%	27.0%	10.0%

*Proportion who have smoked in the last 30 days.

**Table 2:** Means for matching variables

	Sample Size^*^			**Mean (S.D.)**		
**Group**	**Survivor**	**Peer**	**Listed**	**Survivor**	**Peer**	**Listed**
**Variable**						
Age (yrs)^**^	100	44	96	64.90 (10.09)	57.40 (13.9)	64.4 (10.08)
Days of Smoking in the past 30	100	44	96	4.25 (10.3)	6.29 (11.7)	2.54 (8.13)
Lifetime duration of smoking	60	27	53	36.60 (15.0)	30.40(14.2)	28.90 (15.1)
How often do you drink alcohol? (during month)	100	44	95	1.50 (1.63)	1.50 (1.30)	1.64 (1.64)
How much alcohol do you drink on a typical day when you are drinking? (number of drinks)	99	42	95	0.66 (0.70)	0.98 (0.87)	0.77 (0.74)
How often do you drink six drinks of alcohol in one day? (during month)	100	34	95	0.78 (0.95)	1.03 (1.31)	0.82 (0.82)

*: Sample sizes vary due to missing data.

**: Median ages (range) in survivor group, peer group, and listed group were 65.5 (33-90), 59.5 (29-80), and 64.0 (32-90), respectively.

**Table 3:** P-values for matching variables

	Sample Size^*^			**P-Value**		
**Group**	**Survivor**	**Peer**	**Listed**	**Survivor vs. Peer^#^**	**Survivor vs. Listed^$^**	**Quality of Matching^^^^**
**Variable**						
Sex of subject^!^	100	44	96	0.47	1.00	0.99
Age (yrs)^!!^	100	44	96	0.017	0.79	1.00
Education^!^	99	44	96	0.75	0.41	0.018
Employment^!^	100	44	96	0.18	0.25	0.28
Days of smoking in the past 30	100	44	96	0.07	0.23	0.77
Lifetime duration of smoking	60	27	53	0.32	0.011	0.18
How often do you drink alcohol? (during month)	100	44	95	0.77	0.44	0.23
How much alcohol do you drink on a typical day when you are drinking? (number of drinks)	99	42	95	0.40	0.26	0.53
How often do you drink six drinks of alcohol in one day? (during month)	100	34	95	0.51	0.42	0.25

*: sample sizes vary due to missing data.

#: Two-sided p-values for testing H_o_: “the marginal distribution of survivors is the same as that of peers” vs. H_a_: ”the two marginal distributions are different”. The smaller the p-value, the larger the difference between the two marginal distributions.

$: Two-sided p-values for testing H_o_: ”the marginal distribution of survivors is the same as that of listed sample” vs. H_a_: ”the two marginal distributions are different”. The smaller the p-value, the larger the difference between the two marginal distributions.

^^: One-sided p-values for testing H_o_: ”the quality of matching between peers and survivors is the same as that between listed samples and survivors” vs. H_a_: ”the quality of matching between peers and survivors is better than that between listed samples and survivors”.

### Recruitment and retention

Figures 1, 2, and 3 provide flowcharts of the recruitment patterns for each of the three groups. The interviews were conducted over a 6-month time period and the same interviewers were used for each group.

Figure 1 shows the disposition of the recruitment. Eligible survivors were based on those alive at last contact from the Department of Radiation Oncology and confirmed through the Tumor Registry Shands at University of Florida. In response to the letter that was mailed, seven survivors called the toll-free number requesting not to be called. After multiple attempts to obtain a corrected phone number, six names had to be dropped from the list. When the calls were made by the professional call center, 20 individuals refused to participate and 83 either repeatedly requested we call back or the call was never answered. Six survivors had died and 14 were either too mentally or physically compromised to participate in the survey. Four partially completed the survey but were never available to finish the questionnaire. All but six telephone numbers from the original 378 five-year survivors were used to complete the 100 surveys (100/372 = 27%).

Figure 2 shows data regarding recruitment of the peer-nominated group. Of the 100 survivors who completed the questionnaires, 61 recommended at least one peer: thirty-one survivors provided one peer and 30 survivors nominated two peers, yielding a total of 91 nominees. Because we had fallen short of our target of 100 peer nominees, multiple attempts were made to contact all of the nominees; 12 refused and six telephone numbers were non-working. Fourteen nominees reported that they were not eligible, either because they had a history of cancer or were outside the age range. Not shown in the figure, additional analysis showed that the percentage of men and women survivors who nominated a peer were roughly the same. Seventy-two percent of the men were able to nominate at least one peer, as did 79% of the women survivors. Of those peers nominated, we were able to contact 38% of the men nominees compared to 64% of the women. The observed success rate for contacting the peer-nominated group was 44/91 = 48.3%.

The yield for the listed sample as shown in Figure 3 was 101/544 = 19%. Out of the original 980 telephone numbers purchased, all but 436 were used to complete the sample. One hundred seventeen refused with no reason given, and another 188 either repeatedly asked the interviewer to call back or there was no response after multiple attempts. Analysis showed that to complete a sample of women required 4.6 of 10 numbers to be used; men required 5.9. The average of telephone numbers from the strata used to reach the group under 65 years was 5.6 and for those 65 years or older was 5.5.

### Cost of completed calls

The maximum number of telephone call attempts for the survivor, peer nominee, and listed sample individual were 11, 19, and 15, respectively. The rate of completed calls per hour ranged from a high of .65 for the survivors to a rate of .40 for the peer-nominated group (See Table 4). For each completed call in the survivor group, the cost, which included the $25/hour charged by the call center, was $38.46, compared to $41.66 for the listed sample and $62.50 for the peer-nominated sample.

**Table 4:** Cost per selection method

**Recruitment methodology**	**Survivor N = 100**	**Peer N = 44**	**Listed Sample N = 101**
Total costs per group	$3884.46	$2750.00	$4207.66 (includes purchase of telephone list in hourly rate)
Completes per hour	0.65	0.40	0.60
Costs per completed telephone interview	$25/0.65 = $38.46	$25/0.40 = $62.50	$25/0.60 = $41.66

### Matching

Table 2 shows the means, standard deviations, and Table 3 shows the p-values for the comparison between the survivor group versus the peer-nominated and listed sample groups, respectively. Column five in Table 3, titled Survivor vs. Peer, shows the two-sided p-values for testing whether the marginal distribution of the variable in column 1 for survivors is the same as or different than that for the peer-nominated sample. Column six in Table 3, titled Survivor vs. Listed, shows the two-sided p-value for testing whether the marginal distribution of the variable in column 1 for survivors is the same as or different than that for the listed sample. For both columns five and six, the smaller the given p-value, the larger the difference is between the two marginal distributions. The final column in Table 3 shows the one-sided p-value for the test of the overall quality of match. The p-values were obtained by testing the null hypothesis, “the quality of matching between peers and survivors is the same as that between listed samples and survivors” vs. the alternative hypothesis, “the quality of matching between peers and survivors is better than that between listed samples and survivors.”

The peers smoked more in the 30 days prior to the survey (average smoking days 6.29) compared to both the survivors and the listed sample. The difference in past 30-day smoking between survivors and the peer group yielded a two-sided p-value of .07, whereas the two-sided p-value for the survivors and the listed sample was .23. Table 3 shows, however, that the quality of the match at the individual pair level did not differ on days of smoking in the past 30 days (p = 0.77). That is, we could not reject the null hypothesis. In terms of lifetime duration of smoking, there was a trend toward the peer-nominated group yielding a better match with the survivors than the listed sample (p = 0.18). The survivors had significantly greater duration on smoking than the listed sample (36.60 vs. 28.90, p = 0.011), whereas survivors and peers (36.60 vs. 30.40, p = 0.32) were more similar. No differences approached significance for alcohol use.

Overall, we observed that the quality of the matching was not significantly different between the two methods except on age, sex, and education level. Specifically, at the individual pair level, the listed sample produced a better match on age and sex than the peer-nominated group (p = 0.99 and 1.00, respectively). Age (64.90 vs. 64.40) (Table 2) and sex distribution (72% men vs. 71% men) (Table 2) were nearly identical for the survivors and listed sample, respectively. The peer-nominated group produced a better match on education than the listed sample (e.g., 6.8% less than high school for peers vs. 7.0% less than high school for survivors) (Table 1). Thus, as we show in Table 3, we reject the null hypothesis (p = 0.018).

## Conclusions

The key findings from this study are (1) The quality of matching between the two methods of choosing a comparison group for five-year cancer survivors did not differ except for education; the peer method provided a significantly better match for the survivors than the listed sample, and the listed sample, as expected, produced a match on age and sex based on the criteria used to purchase the list; (2) The yield for the listed sample method was greater than for the peer-matching method; (3) The cost per completed interview was greater for the peer-matching method than the listed sample. Previous studies have documented important changes in patients’ ability to function following cancer treatment but the goal of survivorship research has expanded to include understanding the more subtle effects of treatment [25]. To accomplish this goal, longer term studies with appropriate comparison groups are needed [43].

Our results suggest that both methods of selecting a comparison group yield similar matches to the survivor group with the exception of education level. The outcome of our study is somewhat different from that reported by Kaplan et al. [35]. They found that peer nominees were better educated than the target group and hospital patients from the same catchment area. We found that the peer group was less educated. This is an issue that may affect some survivor studies, especially where socioeconomic status is a consideration. We found a trend toward a better match on lifetime duration of smoking for the peer-nominated and survivor groups that may interest some investigators. Smoking exposure is known to carry its own health risks, and when the cancer survivors and controls are not matched on this variable, it may be problematic to separate the effects of cancer treatment from those of smoking exposure.

Kaplan found [35] that older patients were less likely to nominate a peer for the comparison group than younger patients. Our challenge was not in the nomination of a peer but in our ability to contact that nominee, which affected the yield of participants and the cost per completion of the survey. Reducing the delay by telephoning the peers immediately after their number is obtained might reduce the amount of nonworking numbers. A second alternative could be to mail a letter to the nominee asking them to verify their telephone number. These methods would allow immediate corrective actions to any bias in men and women survivors having correct contact information. Our success with the toll-free number for the survivors to call for information suggests that we should offer one to nominees in the future. In short, refining the methodology may provide a closer match on sex for the peer-nominated and survivor group while also lowering the cost per completion. Overall, 91 peers were nominated, but after 19 attempts, we were only able to reach 44 peers. Of those 44 peer respondents, we noted that 19 of the women peers were nominated by women survivors, while 6 were nominated by men survivors. Our instructions to the survivors did not specify that the nominees be the same sex. Visual inspection of the data suggests that the telephone numbers of the male nominees were less accurate or less likely to be answered after multiple attempts than for the women nominees. This added to the cost per completion and may have contributed to the poorer match on sex.

The results of the test for match quality showed the two methods yielded similar results. This must be considered in light of the greater cost per successful completion for the peer-nominated method and its low yield in this study. We maintain that our experience is important for any researcher hoping to investigate the late effects of cancer or cancer treatment among older adults [25]. We found that the survivors nominated peers, but the challenge was in contacting those nominees. Readers should be aware that the listed sample was purchased for $715, and this was a part of the variable cost figured into the survey center’s hourly rate. Mailings to the initial 378 survivors added to our recruitment costs, but are not included in the survey center’s hourly rate.

In summary, we tested the methods using both a test of the marginal distributions by group and the differences at the individual pairs in an attempt to provide methodological evidence for comparison group selection. Our results indicate that investigators of the late effects of cancer treatment should give careful consideration to the methods for comparison group selection. We believe that many of the lessons learned from this study generalize to comparison group selection for the study of other adult-cancer survivors.

## Conflict of interest

This research was partially funded by NCI grant CA111593 and NIDCR grant 1U54DEO19261-01 awarded to H. Logan (PI). The authors have no conflict of interest with the findings from this research.

## Authors’ contributions

HL was responsible for the design and execution of survey, and worked closely with the other contributors in the composition of this manuscript. ST assisted in the conception, design, and execution of the study, and worked closely with other contributors in the composition of this manuscript. MC assisted in the conceptual design, provided the data analysis plan, and oversaw the analysis. GT was responsible for managing participant eligibility, and he worked closely with other contributors in the composition and proofing of this manuscript. WM was responsible for managing participant eligibility, and he worked closely with other contributors in the composition and proofing of this manuscript. CR was responsible for managing participant eligibility, and he worked closely with other contributors in the composition and proofing of this manuscript. All authors read and approved the final manuscript.

## Pre-publication history

The pre-publication history for this paper can be accessed here:

http://www.biomedcentral.com/1471-2288/12/63/prepub
